# Highly malignant soft tissue sarcoma of the extremity with a delayed diagnosis

**DOI:** 10.1186/1477-7819-8-84

**Published:** 2010-09-23

**Authors:** Jong Hoon Park, Chang Ho Kang, Chul Hwan Kim, In Jung Chae, Ji Hun Park

**Affiliations:** 1 Department of Orthopedic Surgery, College of medicine, Korea University, Anam Hospital, Seoul, Korea; 2 Department of Radiology, College of medicine, Korea University, Anam Hospital, Seoul, Korea; 3 Department of Pathology, College of medicine, Korea University, Anam Hospital, Seoul, Korea

## Abstract

**Purpose:**

To evaluate the characteristics of highly malignant soft tissue sarcoma of the extremity with a delayed diagnosis.

**Materials and methods:**

The clinical and radiological characteristics of 18 cases of highly malignant soft tissue sarcomas of the extremity with a delayed diagnosis were determined.

**Results:**

Ten men and eight women of mean age 44.8 years (range, 15-79 years) were included in this study. Seven cases of synovial sarcoma, three cases each of alveolar soft part sarcoma and malignant fibrous histiocytoma, two cases each of highly malignant leiomyosarcoma and myxofibrosarcoma, and one case of clear cell sarcoma were enrolled. Times from tumor detection to diagnosis ranged from 1 to 3 years in most cases; three of the seven synovial sarcoma cases took more than 10 years to diagnose. Of the seven cases of synovial sarcoma, five cases of small, superficial located masses were simply excised without a pre-surgical biopsy. Three cases of alveolar soft part sarcoma showed characteristic T1- and T2-weighted high signal intensities with signal voids in MR images. In addition, one synovial sarcoma patient and one alveolar soft part sarcoma patient showed evidence of calcification on plain radiographs. However, no general characteristic clinical findings were found to be common to the 18 cases.

**Conclusions:**

Contrary to general expectations, some soft tissue tumors that grow slowly are painless, and those that occur in superficial limbs may be highly malignant. Thus, even when a slow growing, painless superficial mass is encountered in a limb, physicians should keep the possibility of highly malignant soft tissue sarcoma in mind.

## Introduction

Soft tissue sarcomas that develop in the limbs and the axial area, even those that are diagnosed early using appropriate methods and are treated adequately, have a 5-year survival rate of between 62 and 84% [1,2]. Early diagnosis is extremely important for the successful treatment of soft tissue sarcoma. However, delayed diagnosis of soft tissue sarcoma is common. The reasons for these delays are variable, and include, slow-growth, no pain, no palpation due to a deep-seated location, and doctor-associated and socioeconomic factors. Because a substantial proportion of soft tissue sarcomas can grow for a long time without pain, they are often misdiagnosed due to the belief held by a large number of clinicians and the general populations that malignant tumors are painful, grow rapidly, and adhere strongly to adjacent tissues, and thus, slow growing, highly malignant soft tissue sarcomas may not be diagnosed, which is likely to result in poor outcomes and become the basis of disputes between physicians and the patient's relatives. Furthermore, because tumor growth rates are subjective, no definition of a slow growing tumor exists in the literature. According to a study by Lawrence et al. [3], approximately 60% of patients with a soft tissue sarcoma knew they had a tumor, and these patients were diagnosed within 6 months of this realization. In this study, we defined a slow-growing tumor as one that patients had been aware of for over a year, and using this criterion, we analyzed highly malignant, slowly growing soft tissue sarcoma cases clinically and radiologically. Here, we describe the characteristics of these tumors and provide a review of the literature.

## Materials and methods

Of 31 cases with a diagnosis of highly malignant (Grade III according to the WHO classification) soft tissue sarcoma of the limb treated at our hospital between July 1997 and December 2008, we analyzed 18 cases in which the delay between patient awareness of the tumor and diagnosis exceeded 1 year. Highly malignant soft tissue sarcoma was diagnosed based on entries in final pathology reports. The male to female ratio of our cohort was 10:8, and mean patient age was 44.8 years (range 15 to 79 years). The characteristics of the 18 cases of highly malignant soft tissue sarcoma were analyzed based on tumor types determined at final diagnosis, times from patient recognition to diagnosis, the anatomical locations of tumors in limbs, depths of tumor locations in tissue (dichotomized as deep or superficial), tumor sizes, and the characteristics of tumors as determined by plain radiography and MRI.

## Results

**Figure 1:**
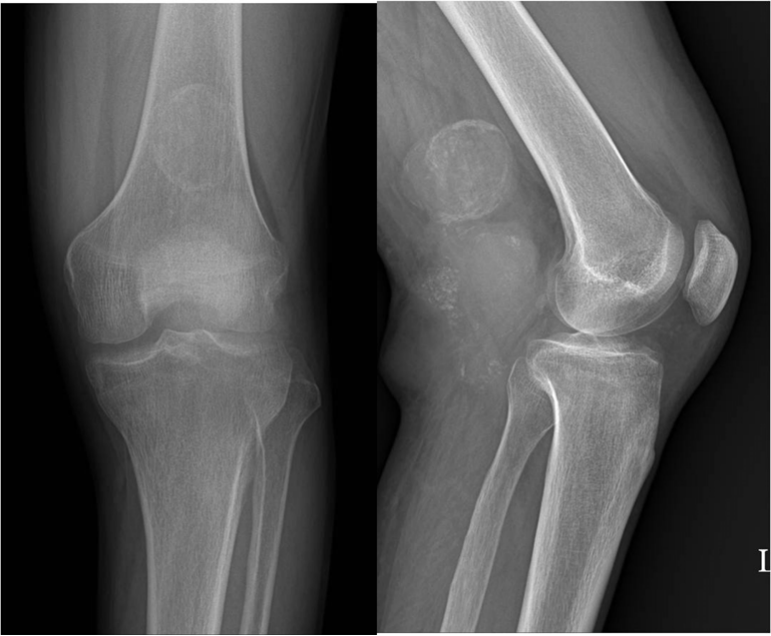
**A plain radiograph showing a well-defined soft tissue mass with marginal calcification posterior to the distal femur**. There is no evidence of associated bone erosion in this patient. This is an example of a synovial sarcoma in one of its more common locations.

**Figure 2:**
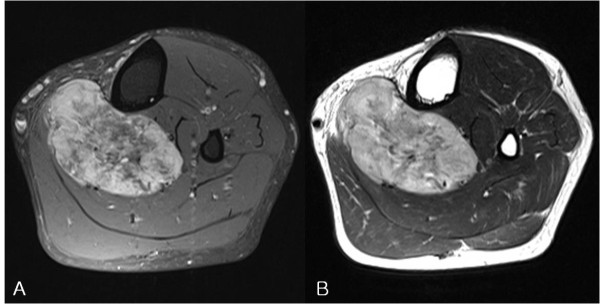
**MRI images of an alveolar soft part sarcoma**. (A) An axial T1-weighted fat-suppressed image and (B) an axial T2-weighted image. High signal on T1FS, T2WI with multiple signal voids are apparent.

Seven cases of synovial sarcoma, three cases each of alveolar soft part sarcoma and malignant fibrous histiocytoma, two cases each of myxofibrosarcoma and high-grade leiomyosarcoma, and one case of clear cell sarcoma were diagnosed. Four of the seven cases of synovial sarcoma, two of the three cases of alveolar soft part sarcoma, two cases of myxofibrosarcoma, and one case each of high-grade leiomyosarcoma and clear cell sarcoma were transferred to our hospital after surgery or biopsy at another hospital. For the remaining 8 cases, all procedures from biopsy to final surgery were performed at our hospital. Affected areas included the thigh (four cases), the lower leg (six cases), the foot and ankle joint (three cases), the forearm (four cases), and the popliteal area (one case). Pulmonary metastasis was detected at diagnosis in all alveolar soft part sarcoma cases and in one clear cell sarcoma case. However, no distant metastasis was detected in the other tumor cases. In six cases, primary tumors were located in a deep region, and in twelve cases, they were located superficially. All three malignant fibrous histiocytoma cases occurred in patients in their 70 s, whereas the alveolar soft part sarcomas occurred in one child (> 10 years old), and in one 20 and one 30 year old. Synovial sarcomas occurred in individuals with ages at onset ranging from the second to the sixth decade, but it occurred preferentially in adults. Delays between tumor recognition by a patient to diagnosis were between 1 and 3 years in most cases. However, in three cases of synovial sarcoma, it took more than 10 years to reach a diagnosis, and in another case of synovial sarcoma, it took more than 5 years. In one case of synovial sarcoma, recurrence occurred at the primary tumor location several times over 20 years. No common characteristic findings were evident by plain radiography, although one case of synovial sarcoma (Figure 1) and one case of alveolar soft part sarcoma showed calcification on plain radiographs. In all three cases of alveolar soft part sarcoma, the characteristic MRI finding was of high signal intensity with signal voiding on T1 and T2 images (Figure 2), which was attributed to the presence of abundant blood vessels. However, no other tumor-specific findings were evident. A diagnostic biopsy was only performed in one of the seven synovial sarcoma cases. In this case, a relatively large tumor was fixed in the superficial layer prior to final operation. However, in the other six cases, simple excision was performed without a pre-surgical biopsy because tumors were small or superficially located. In contrast to synovial sarcoma cases, diagnostic biopsies were performed in all malignant fibrous histiocytoma and alveolar soft part sarcoma cases due to a relatively large tumor size or a deep location.

## Discussion

Some types of soft tissue sarcoma that develop in the limbs or axial skeleton grow slowly over several years, or remain the same size for years or even decades, and then suddenly start to grow. Because these tumors are painless and movable by palpation in many cases, they are often misdiagnosed as benign tumors, and simple excision is performed without adequate pre-surgical evaluation. Importantly, if simple excision is performed without biopsy, the tumor can recur several years later, when all concerned will be taken aback by its aggressive nature. Both physicians and patients have a preconceived idea that malignant tumors are painful and grow rapidly. Furthermore, soft tissue sarcomas are much rarer than benign tumors, and thus, physicians often fail to recommend appropriate tests or perform a comprehensive biopsy. In addition, patients may not even consider consulting a physician in some cases because they do not consider the tumor to be serious. Tumors that do not change or change only slowly over time are often considered benign, but tumor growth rate should not form the basis of a diagnosis. According to Lawrence et al. [3], approximately 50% of soft tissue sarcoma patients are diagnosed at about 4 months after patient recognition, and an additional 20% are diagnosed after 6 months. Furthermore, because the literature provides no precise definition of 'slowly-growing' sarcoma, we considered a slow-growing tumor to be one known to the patient for more than one year prior to diagnosis. Synovial sarcoma is a representative type of slowly growing highly malignant tumor, and it has been reported that in synovial sarcoma cases, a substantial proportion of patients have an average symptomatic period of 2 to 4 years, though in some rare cases, this period has been reported to be longer than 20 years [4]. Of the 18 subjects enrolled in the present study, one case had a symptomatic period of 20 years, and four of the seven synovial sarcoma cases had a symptomatic period of 5-6 years (Table 1). For the reasons listed above, the diagnosis of synovial sarcoma is frequently delayed. In some previous studies, ~20-30% of patients have presented with calcification on plain radiographs [5-7]. Similarly, in our study, calcific density was observed by plain radiography in one case with a tumor in the popliteal area. Sixty percent of soft tissue sarcoma cases occur in the limbs, and the most prevalent affected region is the distal femoral area around the knee joint [8]. Consistent with previous studies, we found that these tumors occurred in individuals with diverse ages ranging from adolescents to adults. To determine the disease stage of soft tissue sarcoma, tumor growth rate and size, and location within tissue should be considered. Characteristics worth noting are that most highly malignant sarcomas are located deep within limb muscles, and that tumor sizes are larger than 5 cm [9]. In the present study, six of seven synovial sarcoma cases were located in superficial areas, but these were small tumors with a long axis of less than 5 cm in four cases. For these reasons, six of the seven were excised without sufficient biopsy or examination. The characteristics of the alveolar soft portions of sarcomas are that they comprise only 0.5-1.0% of soft tissue sarcomas, grow slowly, have metastasized in 20-25% of cases at diagnosis, respond poorly to chemotherapy, and have a poor prognosis [10,11]. Onset usually occurs during adolescence or in young adults, and the only symptom is restriction of joint movement. In our study, one of the three cases of alveolar soft part sarcoma occurred in adolescence, and the other two occurred in young adults. In two of these three cases, the chief complaint was of a painless tumor, whereas the third admitted restricted joint movement with mild pain. At time of diagnosis, pulmonary metastasis was detected in all three cases. In contrast to other soft tissue sarcomas, alveolar soft part sarcomas have characteristic radiologic features, that is, MRI T1- and T2-weighted images have high signal intensities and signal voids due to the presence of abundant blood vessels [12-14]. Accordingly, these tumors can be misdiagnosed as hemangiomas or A-V malformations. In the present study, one case was attributed to hemangioma at initial examination. In alveolar soft part sarcomas, solid tumor tissues are surrounded by vascular tissues and blood flow wash-out is slow. In contrast, A-V malformations are comprised of pure vascular tissue without accompanying tissues, and blood flow wash-out is rapid, which enables the differentiation of these two conditions [14-16].

**Table 1:** Clinical data of all materials

No	Gender	Age (Yrs)	Duration (Yrs)	Diagnosis	Pain	Location	Depth	Size	Previous biopsy
1	M	42	6	S S	No	thigh	deep	12 × 7 × 7	N
2	M	27	20	S S	No	forearm	superficial	3 × 2 × 2	N
3	F	53	2	S S	mild	ankle	superficial	1.8 × 1.2 × 0.5	N
4	F	53	10	S S	mild	foot	superficial	5 × 5 × 6	Y
5	M	15	1	S S	No	ankle	superficial	3 × 3	N
6	F	28	1	S S	mild	forearm	superficial	2 × 2	N
7	M	40	10	S S	Yes	popliteal	superficial	5 × 5	N
8	M	70	1	MFH	No	thigh	superficial	9 × 4	Y
9	F	79	3	MFH	No	forearm	superficial	15 × 7	Y
10	M	69	1	MFH	No	lower leg	superficial	5 × 2	Y
11	F	16	1	Alv SS	mild	thigh	deep	5 × 4 × 3	Y
12	M	27	3	Alv SS	yes	lower leg	deep	7 × 4 × 11	Y
13	M	32	3	Alv SS	yes	lower leg	deep	15 × 7 × 6	Y
14	M	72	3	HGL	No	thigh	deep	4 × 4 × 5	Y
15	F	68	3	HGL	No	lower leg	superficial	5 × 4 × 3	N
16	F	52	2	MFS	mild	lower leg	superficial	14 × 9	Y
17	M	31	1	MFS	mild	lower leg	deep	10 × 5	Y
18	F	50	1	CS	No	forearm	superficial	2 × 3	N

M: male, F: female, S S: synovial sarcoma, MFH: malignant fibrous histiocytoma, HGL: high grade leiomyosarcoma, Alv SS: alveolar soft part sarcoma, CS: clear cell sarcoma, MFS: myxofibrosarcoma

Since most soft tissue sarcomas lack defined radiological characteristics, it is not easy to differentiate sarcomas, even by MRI. Nonetheless, alveolar soft part sarcomas do have specific imaging results, and thus, imaging tests may play a decisive diagnostic role. The general characteristics of most sarcomas are that they grow quickly, are located deep within tissue, and are relatively large. As shown by the present study, the characteristics of some types of soft tissue sarcomas contrast with the traditionally held opinion that they are small slow-growing masses with a superficial location. Fortunately, non-specific soft tissue sarcomas are uncommon, and if preconceived notions are avoided, they can be diagnosed early. Synovial sarcoma cases occur primarily in adults in the vicinity of the knee joint and in the lower leg, and the tumors grow slowly and occur superficially. On the other hand, alveolar soft part sarcomas occur in adolescents and young adults, and are found preferentially around the knee joint and have specific MRI characteristics.

## Conclusions

According to this study, some soft tissue tumors that grow slowly are painless, and some that occur in the superficial limbs may be highly malignant. The most important characteristic of soft tissue sarcomas is their non-specific nature. Thus, even when a slow growing, painless superficial mass is encountered in the limbs, after eliminating absolutely benign possibilities, such as, a ganglion of the wrist or lipoma of the back, physicians must consider the possibility of sarcoma and conduct a careful examination.

## Competing interests

The authors declare that they have no competing interests.

## Authors' contributions

JoHP drafted the manuscript, ChaHK and ChuHK participated in the design of the study, IJC and JiHP conceived of the study, and participated in its design and coordination. All authors read and approved the final manuscript.
